# Further optimization of an ADMA Biologics' proprietary manufacturing process to ensure removal of impurities associated with thrombosis risk in IVIG products

**DOI:** 10.3389/fcvm.2026.1874074

**Published:** 2026-09-24

**Authors:** Kaitlin Kestenberg, Laura Holler, Paul O’Grady, Rebecca Avila, Miranda Capra, Adam Grossman

**Affiliations:** ADMA Biologics, Boca Raton, FL, United States

**Keywords:** Factor XIa, immunoglobulin, primary immunodeficiency, safety, thrombosis

## Abstract

Thromboembolic events (TEEs) are a rare, but serious adverse event for patients receiving immunoglobulin therapy. Patient and product-specific risk factors can increase the risk for these adverse events. To reduce this risk, ADMA has further optimized the manufacturing process of intravenous immunoglobulin (IVIG) products to remove impurities that may precipitate these events. Key attributes were tested in three lots of ADMA's IVIG products from the further optimized process. Across the three lots analyzed, quantification values for each parameter tested were at or below the limit. Since the commercial release of ADMA's IVIG products produced using the further optimized manufacturing process, ADMA's post-marketing pharmacovigilance surveillance and the FDA Adverse Event Reporting System has received zero reported cases of TEEs from July 1, 2025 to July 31, 2026. ADMA's further optimized manufacturing process ensures removal of impurities while also ensuring high recovery of immunoglobulin from source material.

## 1 Introduction

Thromboembolic events (TEEs) are rare, but serious adverse events for patients receiving immunoglobulin (IG) therapy. A retrospective, claims-based study has found an incidence rate of 1% for TEEs occurring within 24 h of IG exposure across IG products (1). Risk factors that pre-dispose patients to these adverse events include advanced age, prolonged immobilization, history of thrombotic events, estrogens, a hypercoagulable state, indwelling central vascular catheters, and cardiovascular risk factors (2, 3). Additional risk factors for TEEs associated with IG products can include procoagulant proteins found in the plasma source for the IG product (2). Naturally occurring procoagulant protein impurities found in plasma are removed through the plasma purification process to safe and established levels to ensure IG product safety (4). Procoagulant impurities include coagulation factors such as activated factor XI (FXIa).

In 2010, a manufacturer initiated a voluntary withdrawal of IG product due to an increase in thromboembolic events (5). Root-cause analysis identified FXIa as the primary impurity associated with the batches of IG products that had the increased reported TEEs (5). In June of 2013, the Food and Drug Administration (FDA) required that the warning for these events be elevated to a Boxed Warning for the entire class of IG products due to the increase in reported TEEs (6, 7). With this increase in reports and changes to labeling requirements by the FDA, some manufacturers have taken additional steps for the removal of impurities that may increase the risk of TEEs, which have includes additional steps in the manufacturing process such as cation exchange chromatography (5, 8).

ADMA Biologics produces two IVIG products, BIVIGAM® (immune globulin intravenous, human 10% liquid) and ASCENIV™ (immune globulin intravenous, human-slra 10% liquid). BIVIGAM and ASCENIV are approved for the treatment of primary humoral immunodeficiency in adults and pediatric patients 2 years of age and older (3, 9). Both are manufactured using similar methodologies, with one distinction in the starting material. BIVIGAM is manufactured using normal source plasma (9). ASCENIV is manufactured using a blend of normal source plasma and plasma from donors with elevated RSV-neutralizing antibodies (4). This tailored plasma pool is achieved through a patented microneutralization assay and results in a unique 10% liquid IVIG with high-titer RSV plasma while maintaining broad humoral coverage (3, 4). In order to improve the IG yield from starting plasma material, ADMA further optimized the manufacturing of its IVIG products. This paper describes ADMA Biologics' approach to ensuring safe levels for FXIa and other important impurities along with a review of adverse events, as they relate ADMA's process to real-world results. The ADMA-IVIG process has been optimized for all unit operations to perform multiple impurity removal, resulting in an efficient process with multiple steps that remove FXIa, IgA, IgM, prekallikrein activator (PKA) and other key impurities.

## 2 Methods

### 2.1 Manufacturing

ADMA IVIG products are licensed products, approved by the FDA and meet requirements for IVIG products. These products are manufactured by a modified Cohn-Oncley fractionation method. The details of ADMA Biologics’ manufacturing process for IVIG products have been previously reported with the systematic quality by design optimization after acquisition of the manufacturing plant from Biotest (4). This process has since then been further optimized with the addition of anion and cation exchange steps for added purification, an increase to a total of six virus inactivation steps to remove or inactivate blood-borne pathogens, and yield improvements resulting in a 20% or more increase in production from the same starting plasma material.

### 2.2 Analytical testing

Key attributes related to procoagulant impurities were measured in three IVIG product lots produced using ADMA's further optimized process. These included Factor XIa, cholesterol, non-activated partial thromboplastin time (NAPTT), PKA, IgA, IgM, and chromatographic purity. Factor XIa enzyme linked immunosorbent assay (ELISA) was used to determine the total FXIa at key process steps and in the final product. NAPTT assay was used to assess procoagulant activity at a 1:10 dilution and followed the European Pharmacopoeia (EP) compendial method with one difference, which is the positive control, which consists of drug product spiked with FXIa to assess system and assay suitability. Cholesterol was measured using a fluorometric assay that measures total cholesterol in human plasma-derived products. PKA activity was determined by an endpoint amidolytic method. Total IgA and IgM presence were determined by ELISA. Chromatographic purity was analyzed using high performance liquid chromatography (HPLC) to determine the relative amount of aggregates, dimers, monomers, and fragments. The percent yield change was assessed across 24 lots of ADMA's IVIG products.

### 2.3 Post-marketing adverse event surveillance

Cases of TEEs were searched via internal ADMA post-marketing pharmacovigilance database and public FDA adverse event reporting system (FAERS) (10). Reports were included any cases since the first commercial lots of ADMA IVIG products were available from the further optimized process, July 1 2025, to July 31, 2026 (11). Internal post-marketing pharmacovigilance search included reports under the Standardized MedDRA Query “Embolic and Thrombotic Events.” Cases from FAERs were included if they were in the United States. Cases from the literature were included if the product was distinctly mentioned.

## 3 Results

Three lots of ADMA's IVIG products that were manufactured using the further optimized process were analyzed. The results reported for each analytical test were from the final product. All results of the critical attributes analyzed were similar or improved in the further optimized process.

### 3.1 Analytical testing

To assess impurities that can increase the risk for TEEs, FXIa and cholesterol were assessed. Across the three lots analyzed, two of the three had levels below the limit of quantification for FXIa, and all three lots had below the limit of quantification for cholesterol (Table 1). The results of the NAPTT assay across the three lots had an average coagulation time of 293.7 s.

**Table 1:** Results from analytical testing of impurities and bioactivity testing using non-activated partial thromboplastin time (NAPTT).

Assay	Lot 1	Lot 2	Lot 3
FXIa	<0.91 mIU/mL^a^	<0.91 mIU/mL^a^	1.2 mIU/mL
Cholesterol	<0.24 mg/dL^a^	<0.24 mg/dL^a^	<0.24 mg/dL^a^
NAPTT 1:10 dilution	294 s	295 s	292 s
PKA	<3 IU/mL	<3 IU/mL	<3 IU/mL
Proteolytic activity	<10 U/L^a^	<10 U/L^a^	<10 U/L^a^
IgA	<43 µg/mL^a^	<43 µg/mL^a^	<43 µg/mL^a^
IgM	0 µg/mL	0 µg/mL	0 µg/mL
Chromatographic Purity—percent aggregates and fragments	0%	0%	0%

a Limit of quantification.

Additional impurities analyzed revealed effective removal of PKA, proteolytic activity, IgA, and IgM across all three lots. Across the three lots analyzed, there was no evidence of aggregates or fragments in the final IVIG product.

Assessment of yield improvement across 24 lots of ADMA's IVIG process can be seen in Figure 1. With this further optimized process, there was an approximate 20% increase in the yield utilizing the same starting plasma.

**Figure 1:**
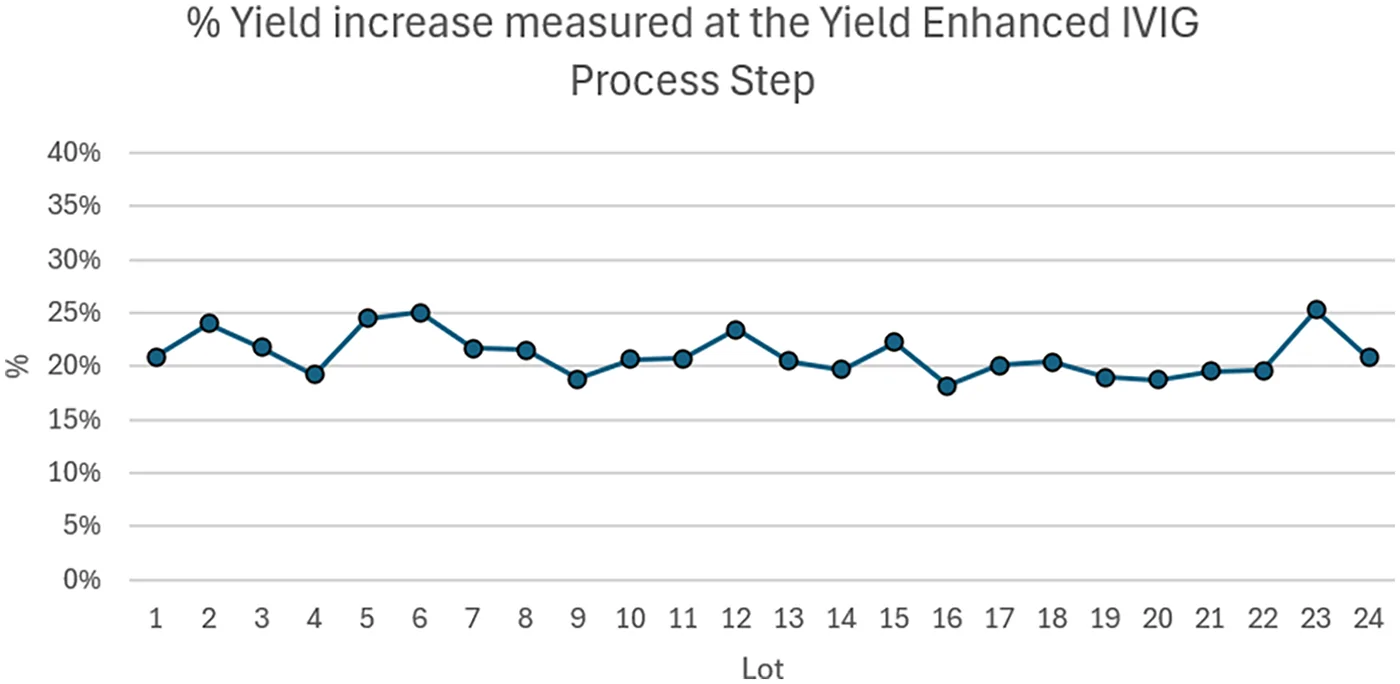
Percent change in yield measured in ADMA's IVIG lots with further optimized process.

### 3.2 Post-marketing adverse event surveillance

Since the commercial release of ADMA's IVIG products produced using the further optimized manufacturing process, ADMA's post-marketing pharmacovigilance surveillance and FAERs has received zero reported cases of TEEs.

## 4 Discussion

Thrombosis is a rare, but serious side effect of IG products, and appropriate manufacturing techniques are needed to ensure safety and quality of the products available, especially for patients with primary humoral immunodeficiency who receive these life-long therapies.

Previous reports on ADMA's manufacturing process demonstrated its ability to remove procoagulant impurities (4). Analyzed lots of ADMA's IVIG products that were produced with the further optimized process yielded results that demonstrate low levels of coagulation factors, with two of the three lots having levels below the limit of quantification for Factor XIa. Additionally, cholesterol, which can also adversely impact patient health over time, was removed below quantifiable limits across all three lots. The results from this further optimized process demonstrate the robust capabilities of ADMA's manufacturing facility to produce an improved product yield while ensuring high quality and safe IVIG products.

FXIa has been linked to thrombotic events in IG products, and removal of this pro-coagulant factor is vital to ensure safety (5). This has previously resulted in improved manufacturing processes, labeling changes, and even the withdrawal and re-introduction of other IG products (6). However, there are additional impurities that can increase the risk for clotting in IG products that also must be taken into consideration. Evaluating NAPTT, a measure of clotting time, allows for an encompassing approach to evaluating thrombotic risk of IG products. Previous studies have demonstrated manufacturing processes resulting in an NAPTT time of >200 s are associated with reduced TEEs (12), while recent studies have demonstrated reduced clotting factors with corresponding NAPTT times around 277 s at a 1:10 dilution (8). In this further optimized process, the average NAPTT time across the three lots at a 1:10 dilution was 293.7 s, with all three lots above 250 s indicating very low levels of procoagulant activity.

ADMA's proprietary manufacturing process minimizes thermal, physical, and chemical stressors on IVIG products as previously described (4). This process has demonstrated its ability to appropriately remove impurities from source material, such as IgA, below quantifiable limits. Though rare, IgA deficient patients have experienced anaphylactic reactions when given IG products that contain moderate amounts of IgA (13). While the majority of IG products contain moderate levels of IgA, a study demonstrating that IVIG-related side effects increase with increasing levels of IgA in the various brands of IVIG has not been conducted and tolerability of individual IVIG products would appear to be dependent on other factors than IgA. Another key indicator of process and product quality in IVIG manufacturing is the quality of the IgG molecules themselves. Across the three lots of ADMA IVIG products analyzed, no aggregates or fragments were detected. This process is designed to prevent the formation of aggregates and does not necessitate additional techniques, such as vibration, to prevent or disrupt aggregation that could weaken the integrity of the IG. This demonstrates not only the sufficient removal of impurities to ensure safety but also preserved quality and integrity of ADMA's IVIG products.

No TEEs have been reported since the release of commercial lots that were manufactured with ADMA's further optimized process. The reports for the post-marketing surveillance is limited by the short follow-up time since the commercial release of these further optimized lots, however this provides supportive real-world context regarding these adverse events. Estimates of thrombosis related to IG products are around 1%, but this risk can vary depending on individual patient's risk factors for TEEs outside of product composition (1, 2, 6). The rarity of these adverse events taken in context with the ability of ADMA's further optimized manufacturing process to reduce impurities known to precipitate these events provides support of the capabilities of this manufacturing process to produce safe IVIG products.

## 5 Conclusion

ADMA's further optimized manufacturing process ensures removal of impurities while also ensuring high recovery of IG from source material. The analytical testing of ADMA's IVIG products after further optimization revealed low Factor XIa and an average NAPTT 293.7 s across three lots, demonstrating that robust measures have been taken in the manufacturing process to reduce the risk of TEEs in patients on ADMA's IVIG products. This process results in IVIG products with high purity, as demonstrated in this analysis and high yield of IG from source materials, reducing the risk of leaving behind valuable antibodies to protect patients against commonly encountered pathogens. These improvements to the manufacturing process can help ensure that patients are able to reliably receive safe and efficacious IVIG products.

## Data Availability

The original contributions presented in the study are included in the article/Supplementary Material, further inquiries can be directed to the corresponding author.
