# Supplementary material for: Further optimization of an ADMA Biologic's proprietary manufacturing process to ensure removal of impurities associated with thrombosis risk in IVIG products
Source: Front Cardiovasc Med. 2026 Sep 24;13:1874074. doi: 10.3389/fcvm.2026.1874074 (PMC13647135; doi:10.3389/fcvm.2026.1874074)
Supplement: Supplementary file 1 [file Supplementaryfile1.docx]

Supplemental Table 1: Critical Line Listings for post-marketing reports of ASCENIV and BIVIGAM for thrombotic events from July 1, 2025-July 31 2026

*Embolic and thrombotic SMQs: Arterial thrombosis, deep vein thrombosis, ischaemic stroke, mesenteric artery thrombosis, pulmonary embolism, retinal visit occlusion, splenic artery thrombosis, splenic infarction, superficial vein thrombosis, thrombosis*

|  | Count of Preferred Term |
| --- | --- |
| Total Events | 0 |

Supplemental Figure 1: Chromatography
